# Residual feed intake phenotype and gender affect the expression of key genes of the lipogenesis pathway in subcutaneous adipose tissue of beef cattle

**DOI:** 10.1186/s40104-018-0282-9

**Published:** 2018-09-20

**Authors:** McKenna Clare, Porter Richard, Keogh Kate, Waters Sinead, McGee Mark, Kenny David

**Affiliations:** 1 Animal and Bioscience Research Department, Teagasc Grange, Dunsany, Meath, C15 PW93 Ireland; 2 School of Biochemistry & Immunology, Trinity College Dublin, Dublin 2, D02 R590 Ireland

**Keywords:** Adipose, Cattle, RFI, *SLC2A4*

## Abstract

**Background:**

Feed accounts for up to 75% of costs in beef production systems, thus any improvement in feed efficiency (FE) will benefit the profitability of this enterprise. Residual feed intake (RFI) is a measure of FE that is independent of level of production. Adipose tissue (AT) is a major endocrine organ and the primary metabolic energy reservoir. It modulates a variety of processes related to FE such as lipid metabolism and glucose homeostasis and thus measures of inter-animal variation in adiposity are frequently included in the calculation of the RFI index. The aim of this study was to determine the effect of phenotypic RFI status and gender on the expression of key candidate genes related to processes involved in energy metabolism within AT. Dry matter intake (DMI) and average daily gain (ADG) were measured over a period of 70 d for 52 purebred Simmental heifers (*n* = 24) and bulls (*n* = 28) with an initial BW±SD of 372±39.6 kg and 387±50.6 kg, respectively. Residual feed intake was calculated and animals were ranked within gender by RFI into high (inefficient; *n* = 9 heifers and *n* = 8 bulls) and low (efficient; *n* = 9 heifers and *n* = 8 bulls) groups.

**Results:**

Average daily gain ±SD and daily DMI ±SD for heifers and bulls were 1.2±0.4 kg and 9.1±0.5 kg, and 1.8±0.3 kg and 9.5±1 kg respectively. High RFI heifers and bulls consumed 10% and 15% more (*P* < 0.05) than their low RFI counterparts, respectively. Heifers had a higher expression of all genes measured than bulls (*P* < 0.05). A gender × RFI interaction was detected for *HMGCS2*(*P* < 0.05) in which high RFI bulls tended to have lower expression of *HMGCS2* than low RFI bulls (*P* < 0.1), whereas high RFI heifers had higher expression than low RFI heifers (*P* < 0.05) and high RFI bulls (*P* < 0.05). *SLC2A4* expression was consistently higher in subcutaneous AT of low RFI animals across gender.

**Conclusion:**

The findings of this study indicate that low RFI cattle exhibit upregulation of the molecular mechanisms governing glucose metabolism in adipose tissue, in particular, glucose clearance. The decreased expression of *SLC2A4* in the inefficient cattle may result in less efficient glucose metabolism in these animals. We conclude that *SLC2A4* may be a potential biomarker for RFI in cattle.

**Electronic supplementary material:**

The online version of this article (10.1186/s40104-018-0282-9) contains supplementary material, which is available to authorized users.

## Background

Increased profitability in livestock production systems can be achieved by reducing the costs of inputs, while maintaining or improving the quantity and quality of outputs [1]. Feed provision accounts for approximately 75% of total variable costs in beef production systems [2]. It is widely accepted that small improvements in FE can have a large influence in the profitability of beef systems, by reducing the overall cost of production [3]. Residual feed intake, first described by Koch et al. [4], can be defined as the difference between an animal’s actual feed intake and that predicted based on the animal’s BW and average daily gain ADG [5]. Efficient cattle, as classified by RFI, are those that consume less feed than expected for a given BW and ADG. Residual feed intake is a moderately heritable trait and considerable genetic variation exists to facilitate selection, making it a prime candidate for biomarker identification [6]. In recent years RFI has been favoured as a measure of FE in beef cattle due to its independence from the production traits used to estimate it [7] and thus the concept is thought to represent inherent differences in basic metabolic processes related to energetic efficiency [1, 2]. Herd and Arthur suggested that approximately one-third of the biological variation in RFI could be attributed to inter-animal differences in digestion, heat increment of feeding, composition of growth and activity, and the remaining two-thirds could be explained by inter-animal variation in energy expenditure [2, 3].

Adipose tissue (AT) is the predominant anatomic site for lipogenesis in ruminants [8] and through its endocrinological activity, modulates a large variety of processes related to feed intake, energy homeostasis, and whole body physiology [9]. While the influence of feed intake on subcutaneous adipose tissue (SCAT) accretion, function and distribution has been widely reported, a lack of knowledge remains on the biological mechanisms mediating the relationship between SCAT and variation in the efficiency of feed utilization amongst individual cattle [10].

**Fig. 1:**
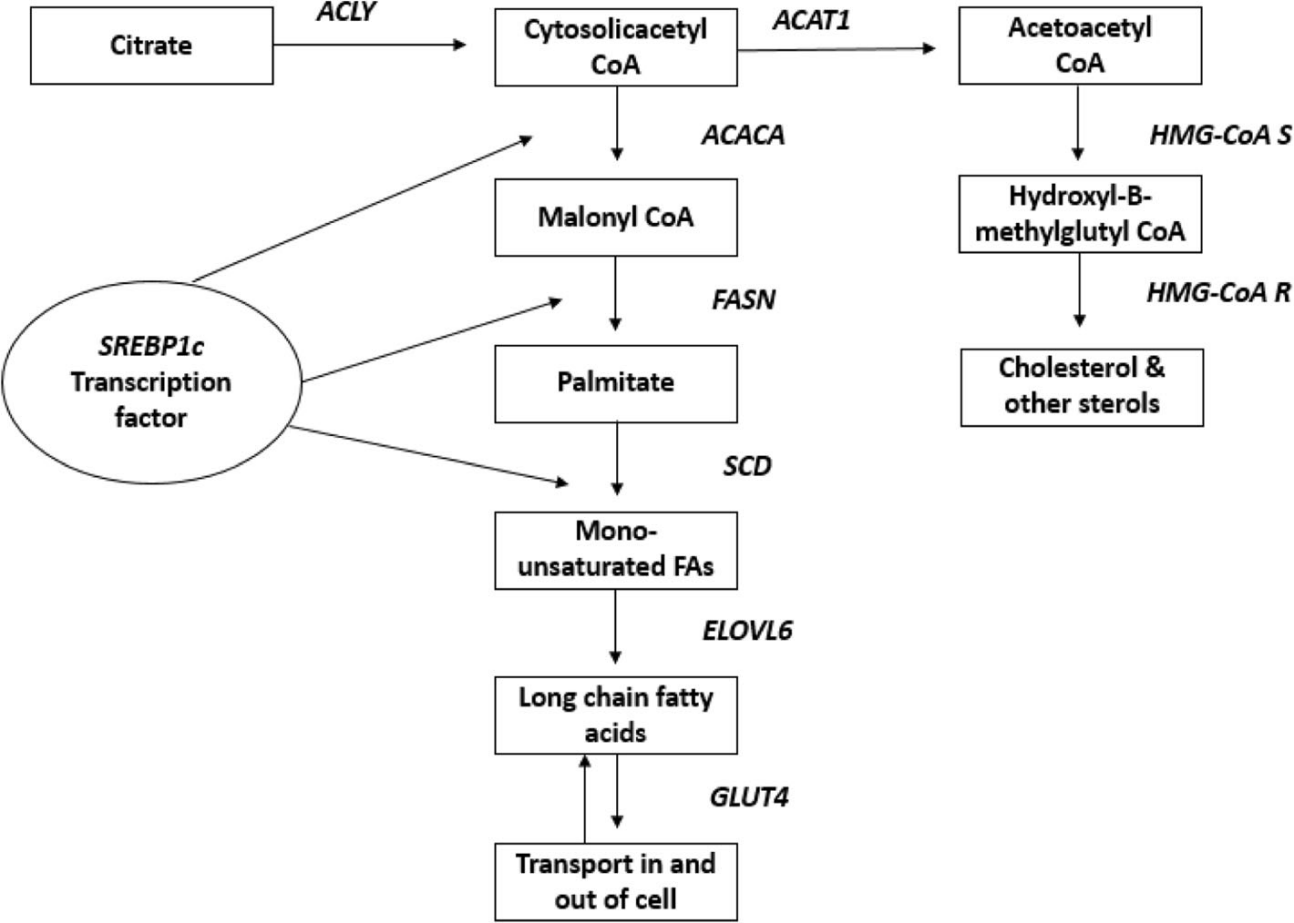
Schematic representation of lipogenesis and cholesterol synthesis pathways. Citrate is cleaved by *ACLY* in the cytosol librerating acetyl CoA. *ACACA* synthesises malonyl CoA from acetyl CoA and malonyl CoA is subsequently converted to palmitate by *FASN*. Following this, *SCD* and *ELOVL6* are responsible for creating long chain fatty acids. Acetyl CoA can also be utilised by *ACAT1* in cholesterol homeostasis pathways which also feature *HMGCS2* and *HMG-CoAR*

A relationship between lipogenesis and RFI in cattle has previously been highlighted [11]. In addition, in a study examining major gene networks associated with RFI, it was observed that RFI was associated with lipid and steroid metabolism, in particular lipogenesis and cholesterol metabolism [12]. Altered lipid metabolism in the liver [13] and muscle [14] has previously been associated with variation in RFI, where it was observed that key genes involved in lipid metabolism were elevated in less efficient cattle. Similarly, Weber et al. [15] concluded that there was a deactivation of the regulatory networks controlling fatty acid metabolism in visceral SCAT of low RFI animals. However, the mechanisms by which lipid metabolism influences RFI status in cattle remain to be elucidated, despite its obvious scientific and economic interest. The current study was undertaken to gain greater insight into the effect of RFI status on the expression of key genes involved in energy mechanisms (see Fig. 1) in SCAT, and how this may be further influenced by gender. We hypothesised that both RFI status and gender would affect the expression of the selected candidate genes.

## Methods

### Animals and management

All procedures involving animals in this study were conducted under an experimental licence (AE1932/P011) from the Health Products Regulatory Authority in accordance with the cruelty to Animals Act 1876 and the European Communities (Amendment of Cruelty to Animals Act 1876) Regulation 2002 and 2005.

The animals used in this study were derived from a purebred herd of Simmental cattle originally established to examine various aspects of the biological control of the RFI trait and which has been well characterised to date in the published literature [4–7, 16]. In order to generate animals for the current study, the 20 highest (inefficient) and 20 lowest (efficient) ranking cows on RFI within the herd, were subjected to a multiple ovulation and embryo recovery programme and bred using artificial insemination to bulls with high and low estimated breeding values (EBVs) for RFI, respectively. Resultant embryos were transferred to crossbred beef heifer recipients. Pregnant heifers were managed under standard protocols and following calving, were allowed to suckle their calves for a period of up to 7 d. In order to standardise rearing, calves were then abruptly weaned. Calves were offered milk replacer (MR) (Blossom Easymix; Volac, Co. Cavan, Ireland) and concentrate in pelleted form using an electronic feeding system (Vario; Foster-Tecknik, Engen, Germany), which recorded all feed-related events including intake of both MR and concentrate, drinking speed, as well as number of rewarded (when calves receive milk) and unrewarded (no milk dispensed) visits to the machine [11]. Calves were subsequently weaned at 10 weeks of age and were offered concentrate and hay on a 50:50 dry matter basis until turnout to pasture at approximately 6 mo of age. Animals grazed high quality pasture under a rotational grazing system. At approximately 15 months of age all cattle were housed within pens of between 5 and 7 animals/pen in a slatted floor shed to facilitate individual feeding using the Calan gate system (American Calan Inc., Northwood, NH). Cattle were fed once daily (08:00 h) and were offered ad libitum access to concentrate (860 g/kg rolled barley, 60 g/kg soya bean meal, 60 g/kg molasses and 20 g/kg minerals/vitamins) and 3 kg grass silage to aid ruminal function. The animals had an acclimatisation period of 14 d to the feeding regime and test facilities before the experimental recording period commenced. The recording period lasted 70 d. Animals were weighed prior to feeding at the beginning and end and weekly throughout the RFI measurement period. Heifers and bulls were ultrasonically scanned by the same person at the beginning and end of the RFI measurement period. A dynamic real-time scanner – (Honda HS 2000 with 2 MHz transducer probe, Honda Electronics Co., Ltd., Toyohashi, Japan) was used to measure fat depth at the third lumbar vertebra, the 13th thoracic rib and the rump (P8 site) on the animal’s right side.

### Computation of traits

While managed the same, heifers and bulls were considered as separate groups for computation of traits and statistical analysis. Average daily live weight gain during the RFI measurement period for each animal was computed as the coefficient (slope) of the linear regression of BW (kg) on time (d) by using the GLM procedure of SAS 9.1 (SAS Inst. INC., Cary, NC). Mid-test metabolic BW (MBW) was represented as BW^0.75^, 35 d before the end of the test which was estimated from the intercept and slope of the regression line. Residual feed intake was calculated for each animal as the difference between actual DMI and expected DMI. Expected DMI was computed for each animal using a multiple regression model, regressing DMI on MBW, ADG and change in back fat (BF). The base model used was:

$$ {Y}_j={\beta}_0+{\beta}_1 MB{W}_j+{\beta}_2 AD{G}_j+{\beta}_3B{F}_j+{e}_j, $$

Where Y_*j*_ is the average of the *j*^th^ animal, *β*_0_ is the regression intercept, *τ*_*i*_ is the fixed effect of the *i*^th^ day, *β*_1_ is the partial regression coefficient for MBW, *β*_2_ is the regression coefficient for ADG, *β*_3_ is the regression coefficient for BF and e_*j*_ is the random error associated with the *j*^th^ animal. The model *R*^2^ coefficient produced from this equation accounted for 0.7 (*P* < 0.001) of the variation in DMI and was used to predict DMI for each animal. Animals were ranked according to RFI within gender and cohorts established representing the lowest (9 bulls and 8 heifers) and highest (9 bulls and 8 heifers) ranking animals on RFI. Power analysis was performed to confirm sample size using software available at www.biomath.info/power/ttest.htm which computed *n*<6 as a sufficient number to yield meaningful results.

### Animal slaughter and sample collection

All animals were slaughtered at an EU licenced abattoir (Eurofarms Foods, Duleek, Co. Meath, Ireland). Animals were weighed prior to slaughter. The mean ± SD bodyweight and age for bulls and heifers at slaughter were 660±67 kg and 18.8±1 months, and 576±38 kg and 18.5±0.9 months, respectively. All surgical instruments used for tissue collection were sterilized and treated with RNase Zap (Ambion, Applera Ireland, Dublin, Ireland). A sample of subcutaneous adipose tissue (SCAT) was collected immediately, from the rump area, after hide removal. Samples were washed with sterile Dulbecco’s Phosphate-Buffered Saline (DPBS; Fisher Scientific, Ireland), cut into small pieces and snap frozen in liquid nitrogen and subsequently stored at − 80 °C until further analysis.

### RNA isolation and purification

Total RNA was isolated from 50 mg of AT using QIAzol (Qiagen, UK). Tissue samples were homogenised in 3 mL of QIAzol reagent using a rotor-strator tissue lyser (Qiagen, UK) and chloroform (Sigma-Aldrich Ireland, Dublin, Ireland). RNA was subsequently precipitated and purified using the RNeasy Plus Universal kit (Qiagen, UK) according to the manufacturer’s guidelines, which included a step to remove any contaminating genomic DNA. The quantity of the RNA isolated was determined by measuring the absorbance at 260 nm using a Nanodrop spectrophotometer ND-1000 (Nanodrop Technologies, Wilmington, DE, USA). RNA quality was assessed on the Agilent Bioanalyser 2100 using the RNA 6000 Nano Lab Chip kit (Agilent Technologies Ireland Ltd., Dublin, Ireland). RNA quality was also verified by ensuring all RNA samples had an absorbance (A260/280) of between1.8 to 2.0 and RIN values of between 8 and 10 were deemed high quality. Any samples that had a (A260/280) absorbance of less than 1.8 were cleaned using a Zymo Research RNA clean & concentrator kit (Cambridge Biosciences, UK). High quality RNA samples were selected for subsequent cDNA synthesis.

### Complementary DNA synthesis

First-strand complementary DNA was synthesised according to manufacturer’s instructions using the High Capacity cDNA Reverse Transcription Kit (Applied Biosystems, Foster City, CA) which utilised Multiscribe reverse transcriptase. Total RNA (2 μg) from each sample was reversed transcribed into cDNA using random hexamers. The converted cDNA was quantified by absorbance at 260 nm, diluted to 50 ng/μL working stocks, and stored at − 20 °C for subsequent analyses.

### Real-time quantitative reverse transcription PCR

Details of primer sets targeting reference and candidate genes, used in this study are shown in Table 1. All primers were obtained from a commercial supplier (Sigma-Aldrich Ireland, Dublin, Ireland). Primer 3 (http://frodo.wi.mit.edu/primer3/) and Primer BLAST (http://www.ncbi.nlm.nih.gov/tools/primer-blast/) software were used to design primers. Primer specificity was assessed using the Basic Local Alignment Search Tool (BLAST) from the national centre for Biotechnology Information. Five genes were tested as reference genes across all samples including: ribosomal protein large PO (*RFLPO*), ubiquitously expressed transcript (*UTX2*), TATA box-binding protein (*TPB*), topoisomerase II-beta (*TOP2B)* and *β-actin.* Relative real time PCR was carried out using the ABI 7500 Fast Real-Time PCR System with Power SYBR® Master Mix (Applied Biosystems, Warrington, UK). Reactions were carried out in a 96-well plate format and prepared in a total volume of 20 μL, with 20–100 ng cDNA, 10 μL Power SYBR® master mix and 1 μL of 5–20 μmol/L forward and reverse primer mix. Optimal cDNA concentration, primer (Table 1) efficiencies and concentrations were determined. Non-template controls were included on every plate for each gene product. To minimize variation, all samples included in each analysis were derived from the same cDNA batch, prepared under the same conditions and samples were run in triplicate. Thermal cycling conditions applied to each assay consisted of an initial Taq activation step at 95 °C for 15 min followed by 40 cycles of 95 °C for 15 s, 60 °C for 60 s, followed by an amplicon dissociation stage (95 °C for 15 s, 60 °C for 1 min, increasing 0.5 °C/cycle until 95 °C was reached). The specificity of the reaction products was also confirmed by dissociation curve analysis. The efficiency of the qPCR reaction was calculated for each gene by creating a standard curve from 2-fold serial dilutions of cDNA concentration. Amplification efficiencies were determined using the formula E = 10^(1/slope), with the slope of the linear curve of cycled threshold (Ct) values plotted against the log dilution of cDNA. Only primers with PCR efficiencies between 80% and 120% were deemed sufficient for subsequent analyses.

**Table 1:** Sequences of bovine oligonucleotide primers used for real-time reverse transcription PCR

Gene symbol	Accession number^a^	Primer sequence (5′ →3′)^b^
*SLC2A4* ^d^	NM_174604.1	F: GGGACTGGTACCCATGTACG
		R: AGGAGGAGTGGCCATAAGGT
*ACAT1* ^d^	NM_001046075.1	F: TCAACGGAGGAGCTGTTTCT
		R: TGCAAATACTGGCAAGACCA
*HMGCoAS* ^d^	NM_001045883.1	F: AGAACGTCTGCCCTCTTTCA
		F: TACAAGGCTGCTGTGTCCAG
*SREBP1c* ^d^	NM_001113302.1	F: CTACATCCGCTTCCTTCAGC
		R: TTCAGCGATTTGCTTTTGTG
*FASN* ^d^	NM_001012669.1	F: TCATCCCCCTGATGAAGAAG
		R: AACTCCACAGGTGGGAACAG
*ACLY* ^d^	NM_001037457.1	F: CAAGAAGGCAGACCAGAAGG
		R: CTGGGCGGTACAGCTTAGAG
*ELOVL6* ^d^	NM_001102155.1	F: GGAAAGCAACGAAAGCTGAC
		R: TGGGTTGTGTGTTTGCTCAT
*SCD* ^d^	NM_173959.4	F: CGACGTGGCTTTTTCTTCTC
		R: GATACCATGGCACGAGTGTG
*ACACA* ^d^	NM_174224.2	F: TGTCCGAAACGTCGATTTTTG
		R: ACGACCTGGTTGCTGTGATAGA
*HMGCoAR* ^d^	NM_001105613.1	F: TGAGGGAGAACATTGCTCGT
		R: ACATGATTTCGAGCTGACGC
*RFLPO* ^c^	NM_001012682	F: CAACCCTGAAGTGCTTGACAT
		R: AGGCAGATGGATCAGCCA
*UTX2* ^c^	NM_001037471	F: TGTGGCCCTTGGATATGGTT
		R: GGTTGTCGCTGAGCTCTGTG
*TPB* ^c^	NM_001075742	F: CCTAAAGACCATTGCACTTCG
		R: CTTCACTCTTGGCTCCTGTG
*TOP2B* ^c^	XM_001254709	F: CCGATGATGATGACGACAAT
		R: TGCTATGGGAGATGCTTTGA
*B-actin* ^c^	U39357	F: CTCACGGAGCGTGGCTACA
		R: GCCATCTCCTGCTCGAAGTC

^a^Accession number in the National Centre for Biotechnology Information database (www.ncbi.nlm.nih/gov/gene)

^b^*F* forward primer, *R* reverse primer

^c^Genes used for as reference for qPCR normalisation; *RFLPO*, Ribosomal protein large PO; *UTX2*, Ubiquitously expressed transcript; *TPB*, TATA box-binding protein; Topoisomerase II-beta TOP2B*, β-actin*

^d^Target genes; *SLC2A4* solute carrier family 2 member 4, *ACAT1* Acetyl-CoA Acetyltransferase 1, *HMGCoAS* Hydroxymethylglutaryl CoA synthase, *SREBP1c* Sterol regulatory element-binding protein, *FASN* Fatty acid synthase, *ACLY* Acetyl citrate lyase, *ELOVL6* Fatty acid elongase 6, *SCD* Stearoyl-CoA desaturase-1, *ACACA* Acetyl-CoA carboxylase, *HMGCoAR* Hydroxymethylglutaryl CoA reductase

### Blood metabolites

Blood samples were obtained by jugular venepuncture from each animal prior to feeding, on d 1 and d 72 of the RFI measurement period. Blood samples were collected into a 9-mL and 4-mL evacuated tube containing lithium heparin and sodium fluoride –tri-potassium (K3) salts of ethylenediaminetetraacetic acid (EDTA) K3, respectively, as anticoagulants (Greiner Vacuette, Cruinn Diagnostics, Dublin, Ireland). Concentrations of albumin, urea, globulin, total protein, β-hydroxybutyrate (BHB), glucose, NEFA, triglycerides and creatinine were determined according to [4]. Globulin concentration was calculated as the difference between total protein and albumin concentrations. All metabolite concentrations were measured on an automatic analyser (AU 400; Olympus, Tokyo, Japan). The sensitivity of each assay was defined as the lowest concentration detectable.

### Statistical analyses

Gene expression data were analysed using GeNorm (GenEx 5.2.1.1; MultiD Analyses, Gothenburg, Sweden). GeNorm is a model based approach software that measures the overall stability of the tested reference genes by calculating the intra-and intergroup CV and combining both coefficients to give a stability value (M-value). The lower the M-value, the higher the stability in gene expression across all samples. An M-value of 1.5 is specified as the default minimum coefficient by the GeNorm program. In the present study, *TBP* and *UTX2* were selected as the most suitably stable reference genes. GenEx 5.2.1.3 was used for efficiency correction of the raw cycle threshold value, normalisation to the expression levels of reference genes and calculation of Ct values for each target gene.

Gene expression data were checked for normality and homogeneity of variance using the UNIVARIATE procedure of Statistical Analysis, Software, version 9.3 (SAS Inst., Cary, NC). Data that were not normally distributed were transformed with the TransReg procedure of SAS 9.3. The genes *SCD*, *ACAT1*, *FASN* and *HMGCS2* were transformed by rising to the power of lambda which was 2, 1.5, 1.5 and 0.5, respectively. Data were further analysed using the mixed model methodology of SAS 9.3 (PROC MIXED, SAS) with treatment and gender, together with their interaction, as the main effects. Calendar day of birth was included in the model as a linear covariate. The R (v3.4) Corrplot package was used to visualise the correlation matrices pertaining to the all candidate genes, RFI and the BF change measurement.

Variables (blood metabolites) having multiple observations, were analysed using repeated measures ANOVA (PROC MIXED procedure of SAS) with the covariance structure as determined by the Bayesian information criterion. Terms for RFI group, sex and sampling day and their interactions were included in the model. Differences in RFI group were determined by *F*-tests using type III sums of squares. The PDIFF option and the Tukey test were applied as appropriate to evaluate pairwise comparisons between RFI group means. The degrees of freedom method used was Kenward Rogers and an unstructured covariance structure was chosen. Data were considered statistically significant when *P* < 0.05 and considered a tendency towards statistical significance when *P* < 0.10.

## Results

### Summary of phenotypic data

The effect of RFI status and gender on DMI, feed efficiency, and performance is shown in Table 2 and slaughter details can be found in Additional file 1. Heifers and bulls had a mean initial BW±SD of 372±39.6 kg and 387±50.6 kg, respectively. During the feed intake recording period heifers and bulls achieved an ADG± SD of 1.2±0.4 kg and 1.8±0.3 kg, and daily DMI±SD of 9.1±0.5 kg and 9.5±1 kg, respectively. Residual feed intake averaged 0.00 kg for both groups and ranged from − 0.47 to 0.6 kg of DM/d for heifers and − 0.50 to 0.59 kg DM/d for bulls. High RFI heifers and bulls consumed 10% and 15% more (*P* < 0.05) than their low RFI counterparts, respectively. Animals of high and low RFI did not differ (*P* > 0.05) in initial BW, final BW or ADG.

**Table 2:** Summary of feed intake and performance data for high and low RFI animals (*n* = 9 bulls, *n* = 8 heifers)

Trait	Bulls			Heifers			*P* ^*d*^		
	High RFI^a^	Low RFI	SD^f^	High RFI	Low RFI	SD	RFI	G^e^	RFI × G
DMI, kg/d	10	8.9	1.08	9.4	8.8	0.6	0.0025	0.28	0.309
RFI, kg DM/d	0.59	−0.5	0.58	0.42	−0.55	0.5	< 0.0001	0.29	0.34
MMBW^0.75^, kg^b^	97.01	97.04	8.64	93.39	93.5	4.06	0.87	0.2	0.79
Initial BW, kg	388.5	382.16	54.91	375.8	364.5	32	0.5	0.62	0.95
Final BW,kg	516.05	508.6	54.28	460.8	458.4	36.1	0.75	0.011	0.82
ADG, kg	1.82	1.8	0.33	1.2	1.3	0.4	0.68	0.001	0.62
Backfatchange^c^, mm	1.45	2.01	0.69	1.17	1.46	0.72	0.18	0.14	0.62

*DMI* Dry matter intake, *RFI* Residual feed intake, *ADG* Average daily gain, *MMBW* Midtest metabolic body weight

^a^High RFI is feed inefficient and low RFI is feed efficient

^b^Metabolic BW^0.75^, kg is determined as BW^0.75^ in the middle of the RFI measurement period which was estimated from the intercept and slope of the regression line after fitting a linear regression line through all metabolic BW (BW^0.75^) observations

^c^Back fat change is mean of fat depth at end*minus* mean of fat depth at start of the feed intake recording period

^d^*P P*-value

^e^*G* Gender

^f^*SD* Standard Deviation

### Real-time quantitative reverse transcription PCR

The effect of divergence in RFI on the expression of genes involved in the lipogenesis pathway in adipose tissue for bulls and heifers is presented in Table 3., There was an RFI × gender interaction for the expression of *HMGCS2* (*P* = 0.03; Additional file 2). High RFI bulls tended to have lower expression of *HMGCS2* than low RFI bulls (*P* = 0.09), whereas high RFI heifers had higher expression than low RFI heifers (*P* = 0.04) and high RFI bulls (*P* = 0.01). There was an effect of gender on the expression of all genes (*P* < 0.05) excluding *ACLY* (*P* > 0.05) in which heifers had a higher expression of all genes compared to bulls.

**Table 3:** Effect of RFI on the expression of lipogenic genes in adipose tissue from cattle divergent for RFI^b^ (*n* = 9 bulls, *n* = 8 heifers)

Gene^b,c^	Bulls			Heifers			*P*-value		
	HRFI^a^	LRFI	SD^e^	HRFI	LRFI	SD	RFI	G^d^	RFI × G
*ACAT1*	2.55	3.88	0.68	3.52	4.38	0.3	0.12	0.0002	0.65
*ACACA*	2.39	2.21	0.48	4.98	6.63	0.31	0.16	< 0.001	0.12
*ACLY*	5.15	4.34	0.29	3.48	3.24	0.54	0.38	0.23	0.32
*ELOVL6*	2.74	2.05	0.43	3.93	3.41	0.41	0.45	0.01	0.62
*FASN*	4.35	4.23	0.35	4.21	5.9	0.39	0.38	0.02	0.23
*SLC2A4*	0.96	1.42	0.33	3.85	5.21	0.52	0.01	< 0.0001	0.34
*HMGCOR*	2.46	1.83	0.39	5.4	6.77	0.81	0.39	0.0002	0.13
*HMGCS2*	1.31	2.1	0.28	6.21	4.9	0.58	0.2	0.01	0.03
*SCD*	4.64	3.65	0.35	6.81	7.6	0.61	0.98	0.003	0.69
*SREBPC1*	2.07	2.45	0.41	4.82	4.55	0.66	0.4	0.0008	0.54

^a^High RFI is feed inefficient and low RFI is feed efficient

^b^Gene expression values were calculated by normalizing cycle threshold (Ct) values for target genes to Ct values of the reference genes after adjustment for efficiencies and interplate variation and converted to values relative to the mean Ct value within each data set using GENEX (MultiD Analyses, AB, Gothenberg, Sweden)

^c^See Table 1 for gene names

^d^*G* Gender

^e^*SD* Standard Deviation

There was an effect of RFI phenotype on the expression of *SLC2A4* (*P* = 0.01) in which low RFI animals had a higher expression than high RFI animals. There was no effect of RFI on expression of *ACACA, ACAT1, ACLY, ELOVL6*, *FASN*, *HMGCR*, *SCD*, and *SREBPC1*. There was no correlation between BF change, RFI and any candidate gene measured (Additional file 3).

### Blood metabolites

Blood metabolites for the high and low RFI groups are presented in Table 4. There were no RFI × sex or RFI × day interactions for any of the metabolites measured. There was a day × sex interaction (*P* < 0.05) for urea where heifers had higher concentrations than bulls on both d 1 and 72. Sampling day had an effect (*P* < 0.05) on all blood metabolites measured, except for BHB and NEFA. RFI status affected (*P* < 0.05) plasma concentrations of NEFA, with concentrations higher in high compared to low RFI animals. Heifers had a higher concentrations (*P* < 0.05) of BHB, Albumin and NEFA than bulls at both samplings.

**Table 4:** Blood metabolites of cattle divergent for RFI

Variable	RFI^c^			G^d^			Day			*P*-value					
	High	Low	SD^e^	Bulls	Heifers	SD	1	72	SD	RFI	Gender	Day	RFI× G	Day × G	RFI × Day
No. of animals	17	17	–	18	16	–	34	34	–	–	–	–		–	–
BHB^a^, nmol/L	0.18	0.22	0.03	0.15	0.25	0.03	0.18	0.21	0.01	0.18	0.017	0.13	0.22	0.06	0.97
Albumin, g/L	34.7	33.7	0.9	32.5	35.9	1.06	31.7	36.7	0.3	0.32	0.006	< 0.0001	0.59	0.86	0.56
Creating umil/L	117.6	118.35	5.7	116.19	119.71	6	110.25	125.75	1.72	0.9	0.56	< 0.0001	0.45	0.7	0.37
Globulin, g/L	40.6	38.5	2.1	40.4	38.7	1.6	38.6	40.5	1.9	0.3	0.4	0.01	0.8	0.5	0.26
Glucose, nmlo/L	4.7	4.8	0.11	4.9	4.6	2	4.5	5	0.11	0.53	0.09	0.0005	0.36	0.17	0.99
NEFA^b^, nmol/L	0.08	0.04	0.017	0.02	0.09	0.018	0.06	0.06	0.006	0.05	0.002	0.18	0.77	0.21	0.35
Total Protein, g/L	75.4	72.2	2.3	73	74.6	2.5	70.3	77.3	0.85	0.2	0.51	< 0.0001	0.73	0.53	0.24
Triglycerides, nmol/L	0.16	0.17	0.03	0.17	0.15	0.03	0.14	0.19	0.009	0.68	0.67	0.001	0.87	0.48	0.93
Urea, nmo/L	3.9	3.7	0.39	3.4	4.17	0.42	5.1	2.55	0.17	0.71	0.13	< 0.0001	0.07	0.01	0.89

^a^*BHB* Beta-hydroxybutyrate

^b^*NEFA* Non esterified fatty acids

^c^*RFI* Residual feed intake βHB

^d^*G* Gender

^e^*SD* Standard Deviation

## Discussion

Lipogenesis is a key process within adipose tissue in response to increased dietary energy intake [8] therefore it seems likely that impaired function of this metabolically important organ could contribute to lower energetic efficiency in cattle. Adipose tissue is composed of a variety of cell types, with adipocytes the most predominant. It acts as an energy depot to maintain metabolic homeostasis, ensuring a rapid response to modifications in nutrient availability [9]. It has also been proposed that adipose cell size may be involved in the regulation of feed intake [10]. Furthermore, fat partitioning between depots changes greatly with the physiological maturity of cattle with SCAT representing the largest proportion of total body fat [12]. In addition, the energetic cost of laying down SCAT is high, impacting overall energetic efficiency and profitability of cattle. Thus coupled with the fact that SCAT is the most readily measured fat depot, the current study refers to experiments undertaken only in SCAT.

The objectives of this study were to examine the premise that differences in the expression of key genes involved in energy expenditure in SCAT contribute to variation in RFI status amongst cattle. While currently there is a dearth of published information on the relationship between the lipogenesis pathway in AT and RFI, our findings add new knowledge on the molecular mechanisms underpinning this trait. To the author’s knowledge, this is the first study to examine the combined effects of RFI status and gender on mRNA expression of key genes in adipose tissue of cattle. Genomic selection models do not differentiate on the basis of gender and, if biomarkers are to be of any scientific utility, it is important to determine whether biological processes regulating economically important traits such as RFI are consistent across gender. The main findings of this study were that: firstly, and not unexpectedly, heifers had a higher expression of lipogenesis related genes than bulls where similar genetics, rearing and finishing conditions prevailed. Secondly, we detected a gender × RFI interaction for observed differential gene expression of *HMGCS2.* Finally, we concluded that divergence for RFI across gender affects the blood plasma concentration of NEFA and the gene expression of *SLC2A4*, indicating that the inefficient animals are more insulin resistant than their efficient counterparts.

It is important to mention that in the current study BF change was included as a variable in the model to predict feed intake, therefore equalising the groups for this trait. Studies have reported that RFI is linked with fat deposition, stating that animals with favourable phenotypes for RFI have leaner carcasses [15], which can lead to unfavourable carcass changes [13, 14] It is important to correct for BF when selecting for RFI to avoid potential negative effects of co-selecting for leaner animals [17]. In addition, equalising the groups for BF within gender in the current study allowed for examination of the relative metabolic functionality of the SCAT and its potential variation with respect to RFI over and above subcutaneous-fat deposition.

The results of the current study indicated higher mRNA expression of nine key genes in the lipogenesis pathway in heifers compared to bulls. This result is not surprising as gender differences in adipose deposition and function have been widely documented in the literature, across multiple species [18–20]. In fact, female cattle accumulate more fat than males at the same age [12, 21, 22] and it has been observed that heifers have larger adipocytes than bulls of the same BW [12, 20]. We observed a numerically higher deposition of BF in heifers compared to bulls and this observation supports the increased expression of lipogenic genes in heifers. The results of the present study are in agreement with Equinoa et al. [20] who observed increased activity of lipogenic enzymes in SCAT in heifers compared to bulls. The comparison between heifer and bull data also provides additional reassurance of our capacity to detect differences in gene expression between the RFI phenotypes should they exist.

Studies have found difference in the genes *ACAT1, ACACA, ACLY, HMGCR, FASN, SCD, ELOVL6* and *SREBP1c* between low and high RFI cattle, in which there was a decrease in gene expression of the regulatory network controlling fatty acid metabolism in adipose tissue of low RFI animals [23, 24]. While this is in agreement with the hypothesis of low RFI animals having less fat deposition, the present study we did not see a difference in expression levels of these genes between animals divergent for RFI. This discrepancy between studies could be explained by varying levels of divergence for RFI in the animals and different breed types and genders etc. employed between studies. Furthermore, the current study failed to detect gender x RFI interactions for gene expression differences which we suspect is due to the different stages of maturity and physiology of the two genders.

3-Hydroxy-3-methylglutaryl-CoA synthase 2*(HMGCS2)* is a mitochondrial enzyme that produces HMG-CoA for ketogenesis, in turn stimulating fatty acid oxidation [25]. In this study we observed a gender x RFI interaction for the expression of *HMGCS2* where high RFI bulls tended to have lower expression of *HMGCS2* than low RFI bulls whereas high RFI heifers had higher expression than low RFI heifers and high RFI. These findings suggest that efficient bulls may have an increase in fatty acid oxidation, suggesting that these animals could be directing their metabolism towards alternative substrate partitioning and fatty acid breakdown in order to facilitate their lower feed intake. However, in the case of the efficient heifers there appeared to be a decrease in fatty oxidation. The reason for the differences observed between genders is unclear, but could be attributed to the different stages of maturity and physiology of the two genders.

The insulin-responsive glucose transporter 4 (*SLC2A4*) plays a key role in insulin-stimulated glucose endocytosis and exocytosis in adipose tissue by rapidly translocating from intracellular storage sites to the plasma membrane [26]. Normally *SLC2A4* gene expression s increased in in response to states of increased metabolic glucose demand. Impaired regulation of *SLC2A4* gene expression and function is associated with insulin resistance and conditions such as type 2 diabetes and obesity in humans and equines [27, 28]. Decreased expression of *SLC2A4* in adipose tissue in human obesity and type 2 diabetes has been observed [29, 30]. Furthermore, *SLC2A4* expression appears to be upregulated in pigs that are mildly under nourished [31] but downregulated in lactating goats [32] . Perhaps the most important finding from this study was that there was an effect of RFI across gender on the expression of *SLC2A4* in which low RFI animals exhibited a higher expression of this gene compared to their high RFI counterparts. This finding indicates that the decreased expression of *SLC2A4* in the high RFI animals represents less efficient glucose homeostasis and increased insulin resistance in these animals. In addition, Kelly et al. [33] reported greater levels of circulating plasma insulin in low RFI animals which supports our findings of increased *SLC2A4* in the low RFI phenotype. Furthermore, because changes in *SLC2A4* expression can alter many aspects of energy utilization it is likely that *SLC2A4* is contributing significantly to variation in RFI.

Measuring the systemic concentration of metabolites can contribute to the characterisation of the metabolic state of animals divergent for phenotypic RFI [4]. Metabolites are typically influenced by environmental factors such as diet type and physiological status and perhaps even animal genotype. In general, there was little evidence for a meaningful effect of either sex or RFI status on the systemic concentrations of metabolites measured. While day of sampling affected the concentration of a number of metabolites this was generally consistent with the improved nutritional status of the animals following 70 d of free access to an energy dense diet. In the current study, high RFI animals had twice the concentrations of plasma NEFA than their low RFI contemporaries. High plasma NEFA levels are the hallmark of insulin resistance in that, elevated NEFA can block the action of the glucose transporter *SLC2A4* which may explain the lower expression of *SLC2A4* observed in the inefficient animals in the current study.

## Conclusion

Our work together with that of [11, 14] indicate that physiological processes in adipose tissue such as lipogenesis, cholesterol metabolism and overall lipid metabolism may be contributing to variation in RFI. Discrepancies between the results reported here and those of other authors may be a consequence of variation in diet, gender, breed and stage of maturity of the animals employed. This study highlights the relationship between important metabolic molecules, *SLC2A4,* plasma NEFA and RFI which is consistent across gender, suggesting that inefficient animals may be insulin resistant when compared to their efficient counterparts. Our findings also suggest *SLC2A4* as a potential robust biomarker for RFI. Future work, including global gene expression profiling and targeted proteomics is warranted to investigate this hypothesis further.

## Additional files

Additional file 1: Slaughter and carcass details for animals divergent for RFI. (DOCX 13 kb)

Additional file 2: RFI x Gender interaction for *HMGCS2* in animals divergent for RFI. (DOCX 45 kb)

Additional file 3: Correlogram highlighting the correlation between all adipose tissue candidate genes from adipose tissue, Backfat measurements and RFI. Visual representation of the correlation matrix between RFI, Backfat change and all genes measured Blue circles (correlation value 1) indicate a positive correlation while red (correlation value − 1) indicate a negative correlation. (DOCX 296 kb)
