# Correction to: Residual feed intake phenotype and gender affect the expression of key genes of the lipogenesis pathway in subcutaneous adipose tissue of beef cattle

**DOI:** 10.1186/s40104-018-0300-y

**Published:** 2018-11-07

**Authors:** Clare McKenna, Richard K. Porter, Kate A. Keogh, Sinead M. Waters, Mark McGee, David A. Kenny

**Affiliations:** 1 Animal and Bioscience Research Department, Teagasc Grange, Dunsany, Meath, C15 PW93 Ireland; 2 School of Biochemistry & Immunology, Trinity College Dublin, Dublin 2, D02 R590 Ireland

## Correction

In the original publication of this article [1], some errors in Table 4 need to be corrected as below:

“Creating, umil/L” should be “Creatine, umol/L”

“Glucose, nmlo/L” should be “Glucose, nmol/L”

“Urea, nmo/L” should be “Urea, nmol/L”

The original publication has been corrected.
