# Supplementary material for: Residual feed intake phenotype and gender affect the expression of key genes of the lipogenesis pathway in subcutaneous adipose tissue of beef cattle
Source: J Anim Sci Biotechnol. 2018 Sep 20;9:68. doi: 10.1186/s40104-018-0282-9 (PMC6146607; doi:10.1186/s40104-018-0282-9)
Supplement: Supplementary file 1 — Slaughter and carcass details for animals divergent for RFI. (DOCX 13 kb) [file 40104_2018_282_MOESM1_ESM.docx]

**Additional File 1.** Slaughter and carcass details for animals divergent for RFI

| RFI^1^ | Gender | Liver weight (kg) | SD^2^ | Kill weight(kg) | SD | Cold weight (kg) | SD | Kill age (months) | SD |
| --- | --- | --- | --- | --- | --- | --- | --- | --- | --- |
| **HRFI** | Heifers | 7.12 | 1.14 | 578.67 | 35.89 | 309.96 | 22.33 | 18.89 | 0.87 |
| **LRFI** | Heifers | 7.15 | 0.9 | 573.2 | 40.03 | 306.02 | 24.57 | 18.3 | 0.9 |
| **HRFI** | Bulls | 7.68 | 1.2 | 659.14 | 49.3 | 365.15 | 27.76 | 19.09 | 0.79 |
| **LRFI** | Bulls | 7.66 | 0.81 | 621.75 | 68.85 | 342.95 | 42.32 | 18.63 | 1.22 |

^1^ HRFI = inefficient, LRFI = efficient

^2^ SD = Standard Deviation
