# Supplementary material for: Residual feed intake phenotype and gender affect the expression of key genes of the lipogenesis pathway in subcutaneous adipose tissue of beef cattle
Source: J Anim Sci Biotechnol. 2018 Sep 20;9:68. doi: 10.1186/s40104-018-0282-9 (PMC6146607; doi:10.1186/s40104-018-0282-9)
Supplement: Supplementary file 2 — RFI x Gender interaction for HMGCS2 in animals divergent for RFI. (DOCX 45 kb) [file 40104_2018_282_MOESM2_ESM.docx]

*

*

*

NS

*

n=8

n=8

n=9

**Additional file 3. RFI x gender interaction for *HMGCS1* in animals divergent for RFI**. HRFI = High RFI; LRFI = Low RFI; H= Heifer; B = Bull. There was an RFI × gender interaction for the expression of *HMGCS1.* High RFI bulls tended to have lower expression of *HMGCS1* than low RFI bulls, whereas high RFI heifers had higher expression than low RFI heifers and high RFI bulls. * denotes significance between groups, NS denotes non-significance.
