# Supplementary material for: Residual feed intake phenotype and gender affect the expression of key genes of the lipogenesis pathway in subcutaneous adipose tissue of beef cattle
Source: J Anim Sci Biotechnol. 2018 Sep 20;9:68. doi: 10.1186/s40104-018-0282-9 (PMC6146607; doi:10.1186/s40104-018-0282-9)
Supplement: Supplementary file 3 — Correlogram highlighting the correlation between all adipose tissue candidate genes from adipose tissue, Backfat measurements and RFI. Visual representation of the correlation matrix between RFI, Backfat change and all genes measured Blue circles (correlation value 1) indicate a positive correlation while red (correlation value − 1) indicate a negative correlation. (DOCX 296 kb) [file 40104_2018_282_MOESM3_ESM.docx]

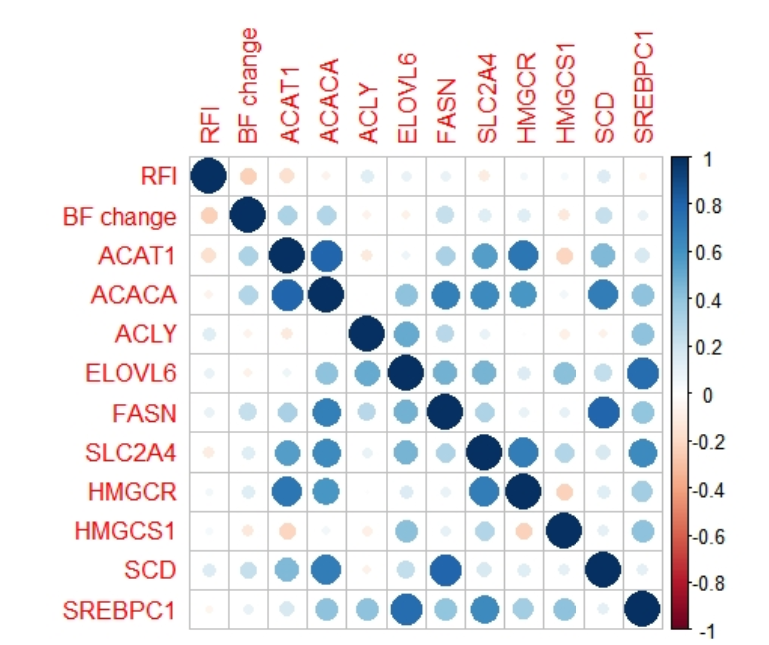


**Correlogram highlighting the correlation between all adipose tissue candidate genes, Backfat measurements and RFI.** Visual representation of the correlation matrix between RFI, Backfat change and all genes measured. Blue circles (correlation value 1) indicate a positive correlation while red (correlation value -1) indicate a negative correlation.
